# Factors controlling bacteria and protists in selected Mazurian eutrophic lakes (North-Eastern Poland) during spring

**DOI:** 10.1186/2046-9063-9-9

**Published:** 2013-04-08

**Authors:** Krystyna Kalinowska, Adam Guśpiel, Bartosz Kiersztyn, Ryszard J Chróst

**Affiliations:** 1 Polish Academy of Sciences, Centre for Ecological Research, Hydrobiological Station, ul. Leśna 13, Mikołajki, 11-730, Poland; 2 Department of Microbial Ecology, Institute of Botany, Faculty of Biology, University of Warsaw, ul. Miecznikowa 1, Warsaw, 02-096, Poland

**Keywords:** Bacteria, Metabolic activity, Protists, Eutrophic lakes

## Abstract

**Background:**

The bottom-up (food resources) and top-down (grazing pressure) controls, with other environmental parameters (water temperature, pH) are the main factors regulating the abundance and structure of microbial communities in aquatic ecosystems. It is still not definitively decided which of the two control mechanisms is more important. The significance of bottom-up versus top-down controls may alter with lake productivity and season. In oligo- and/or mesotrophic environments, the bottom-up control is mostly important in regulating bacterial abundances, while in eutrophic systems, the top-down control may be more significant.

**Results:**

The abundance of bacteria, heterotrophic (HNF) and autotrophic (ANF) nanoflagellates and ciliates, as well as bacterial production (BP) and metabolically active cells of bacteria (CTC, NuCC, EST) were studied in eutrophic lakes (Mazurian Lake District, Poland) during spring. The studied lakes were characterized by high nanoflagellate (mean 17.36 ± 8.57 × 10^3^ cells ml^-1^) and ciliate abundances (mean 59.9 ± 22.4 ind. ml^-1^) that were higher in the euphotic zone than in the bottom waters, with relatively low bacterial densities (4.76 ± 2.08 × 10^6^ cells ml^-1^) that were lower in the euphotic zone compared to the profundal zone. Oligotrichida (*Rimostrombidium* spp.), Prostomatida (*Urotricha* spp.) and Scuticociliatida (*Histiobalantium bodamicum*) dominated in the euphotic zone, whereas oligotrichs *Tintinnidium* sp. and prostomatids *Urotricha* spp. were most numerous in the bottom waters. Among the staining methods used to examine bacterial cellular metabolic activity, the lowest percentage of active cells was recorded with the CTC (1.5–15.4%) and EST (2.7–14.2%) assay in contrast to the NuCC (28.8–97.3%) method.

**Conclusions:**

In the euphotic zone, the bottom-up factors (TP and DOC concentrations) played a more important role than top-down control (grazing by protists) in regulating bacterial numbers and activity. None of the single analyzed factors controlled bacterial abundance in the bottom waters. The results of this study suggest that both control mechanisms, bottom-up and top-down, simultaneously regulated bacterial community and their activity in the profundal zone of the studied lakes during spring. In both lake water layers, food availability (algae, nanoflagellates) was probably the major factor determining ciliate abundance and their composition. In the bottom waters, both groups of protists appeared to be also influenced by oxygen, temperature, and total phosphorus.

## Background

The existence of specific periods of phytoplankton development, such as spring phytoplankton peak and its collapse, clear-water phase, and summer phytoplankton bloom, are characteristic features of lake ecosystems. These three regularly occurring ecological events differ distinctly in many aspects such as water temperature, nutrient availability, quality and quantity of organic matter, abundance and taxonomic composition of phytoplankton and zooplankton, and the importance of two, bottom-up and top-down, control mechanisms in the ecological regulation of planktonic assemblages. Literature data show that these events differ also distinctly in the abundance and composition of protists and their role in controlling bacterial production [1,2] as well as in activity rates of bacteria [3]. For instance, during the spring phytoplankton period, heterotrophic nanoflagellates are the dominant bacterivores and may consume a significant part of the total bacterial production per day [1]. The clear-water phase is the period with a negligible role of protists in bacterial consumption whereas the summer-fall period clearly indicates the dominant role of ciliates as bacterivores [1].

The spring phytoplankton bloom is a specific period that is especially interesting and important for the seasonal successions of planktonic events in lakes [4]. As shown by Zdanowski [5], spring concentrations of total phosphorus determine summer chlorophyll content in lakes. At this time, different groups of organisms, usually reaching maximal abundances, are regulated by different factors. In the spring, phytoplankton maxima in temperate, deep lakes are commonly controlled by physical (light availability) and chemical (nutrient concentrations) factors and only indirectly by temperature. A peak of zooplankton could be the result of algal breakdown if the zooplankton feed on the increased abundances of attached-bacteria decomposing the algal debris [4,6,7]. In spring, protists, mainly ciliates, can build up to 60% of the total zooplankton biomass [8] and are considered to be major consumers of spring phytoplankton blooms, leading to the clear-water phase [9-11].

Numerous studies have been performed to evaluate the effect of lake water trophic status on bacteria e.g. [12-14] and protists [8,15,16], which have indicated an increase of both their numbers and biomass along the trophic gradient. Most of these studies were performed during summer thermal stratification or data for spring and summer were considered together. In addition, little information is available on different aspects of the cellular activity of bacterial communities along the eutrophication gradient, especially during spring.

The aim of this study was to examine abiotic (total phosphorus – TP, total nitrogen – TN, dissolved organic carbon – DOC) and biotic (chlorophyll *a*, bacteria, autotrophic and heterotrophic nanoflagellates, ciliates) factors that may influence the microplankton community, especially the metabolically active fraction of bacteria during spring in different eutrophic Mazurian lakes and water depths.

## Results

### Physical and chemical characteristics of investigated lakes

**Figure 1:**
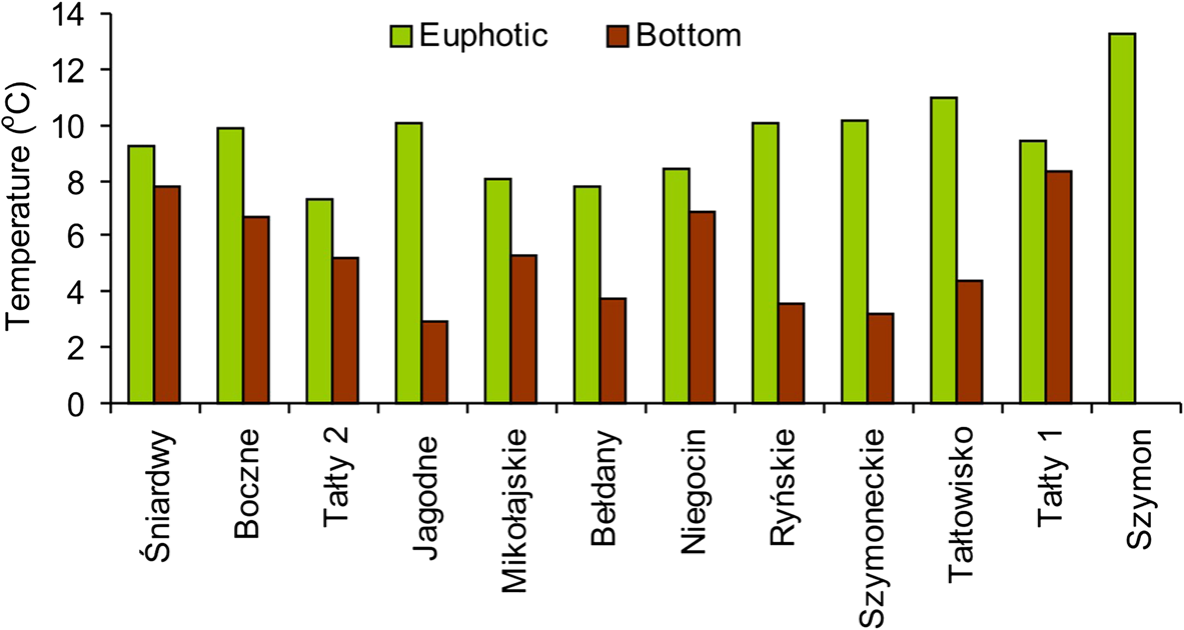
Water temperature in the euphotic zone (green columns) and bottom waters (brown columns) of the studied Mazurian lakes.

**Figure 2:**
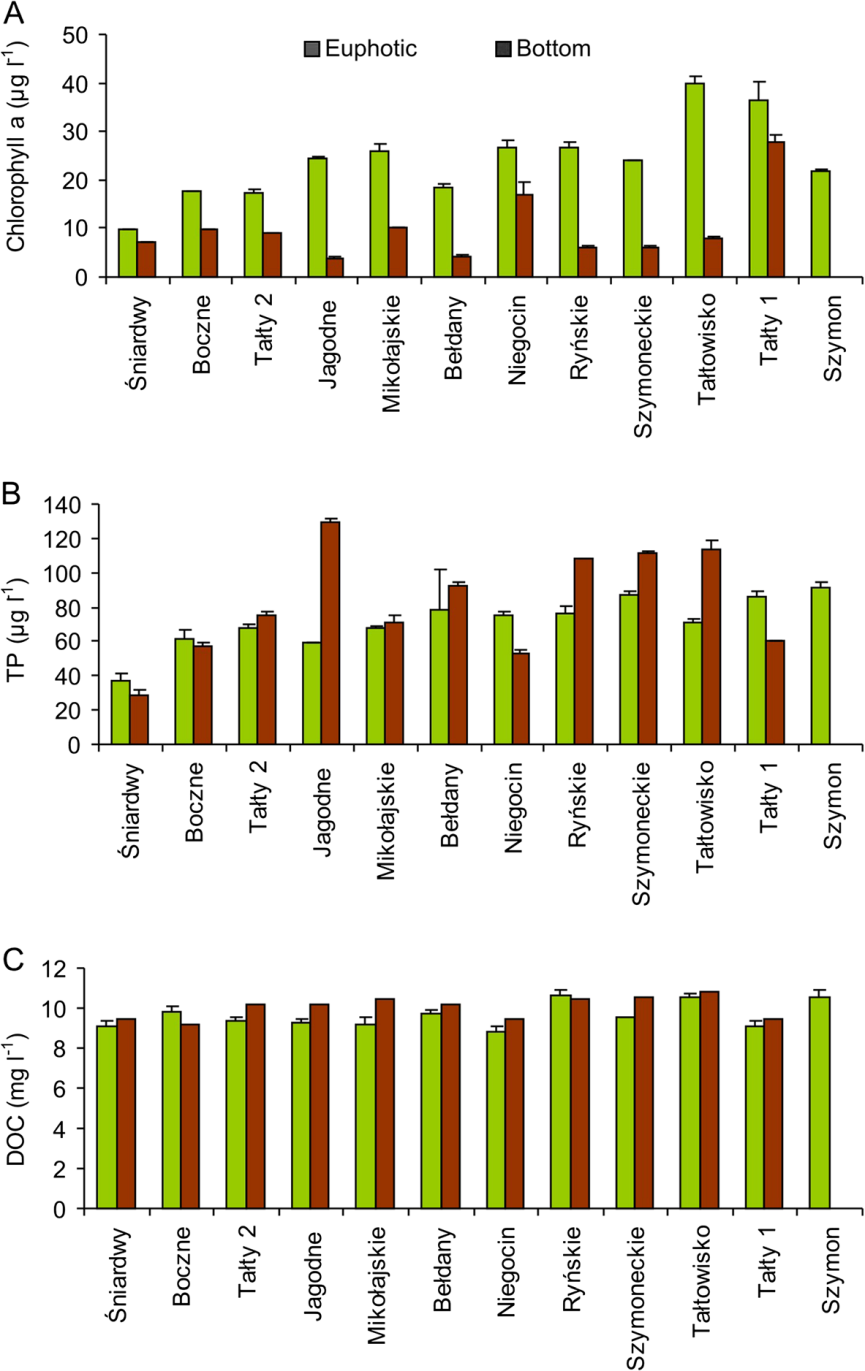
**Concentrations of (A) chlorophyll *a*, (B) total phosphorus (TP) and (C) dissolved organic carbon (DOC) in the euphotic zone (green columns) and bottom waters (brown columns) of the studied Mazurian lakes (mean values and standard deviations of triplicate measurements).**

During the study period, water temperatures in the euphotic and bottom water layers of the studied lakes ranged from 7.3 to 13.3°C and from 2.9 to 8.3°C, respectively (Figure 1). The oxygen concentration varied between 11.4 and 17.3 mg l^-1^ in the euphotic zone and between 2.7 and 13.9 mg l^-1^ in the bottom waters. The pH values were 7.4–8.0 in the euphotic zone whereas slightly lower values were observed in the bottom waters of lakes (6.6–7.5). The conductivity was very similar in the euphotic zone (247 to 335 μS cm^-1^) and in the bottom water layer (242 to 291 μS cm^-1^). Statistically, differences in physical parameters, except conductivity, between the studied layers were found to be significant (Mann–Whitney *U*-test, *p* < 0.001). The Secchi disc visibility depth ranged from 1.1 m (Lake Szymon) to 2.7 m (Lake Śniardwy) (Table 1). Thus, the euphotic zone varied between 2.2 and 5.4 m in the studied lakes. Chlorophyll *a* concentrations were 2–6-times higher in the euphotic zone (17.3 ± 0.8 μg l^-1^ – 39.8 ± 1.6 μg l^-1^) than in the bottom waters (3.8 ± 0.2 μg l^-1^ – 28.0 ± 1.5 μg l^-1^) (Figure 2A). The lakes exhibited different nutrient concentrations. Also, concentrations of total phosphorus (TP) in the euphotic zone varied between 37 ± 4 μg P l^-1^ and 91 ± 3 μg P l^-1^, while they were between 28 ± 4 and 130 ± 2 μg P l^-1^ in the bottom waters (Figure 2B). In both water layers, the lowest values were noted in the meso-eutrophic Lake Śniardwy. In the euphotic zone, total nitrogen (TN) concentrations were more or less similar in the studied lakes (1.05 ± 0.09 – 1.76 ± 0.06 mg N l^-1^), except for Lake Bełdany, where a concentration of 0.79 ± 0.07 mg N l^-1^ was noted (data not shown). Dissolved organic carbon (DOC) concentrations did not differ markedly between the studied lakes and sampling water layers (8.8 ± 0.3 – 10.7 ± 0.2 mg C l^-1^ in the euphotic zone and 9.2–10.8 mg C l^-1^ in the bottom waters) (Figure 2C). The differences between both layers were only statistically significant for chlorophyll *a* concentrations (*p* < 0.01).

**Table 1:** Morphometric and trophic characteristics of the studied Mazurian lakes

**Lake**	**Area (ha)**	**Mean depth (m)**	**Max. depth (m)**	**SD (m)**	**TSI**	**Trophic status**
Śniardwy	11,340	5.8	23.4	2.7	51.5	Meso-eutrophy
Boczne	183	8.4	25.0	2.2	56.9	Eutrophy
Tałty site 2	1,160	13.5	44.7	2.0	57.9	Eutrophy
Jagodne	420	8.7	37.4	1.7	59.1	Eutrophy
Mikołajskie	498	11.2	25.9	1.9	59.5	Eutrophy
Bełdany	941	10.0	46.0	1.6	59.8	Eutrophy
Niegocin	2,600	9.9	39.7	1.9	60.0	Eutrophy
Ryńskie	671	13.5	50.8	1.6	60.9	Eutrophy
Szymoneckie	523	8.7	28.5	1.6	61.2	Eutrophy
Tałtowisko	327	14.0	39.5	1.4	62.5	Eutrophy
Tałty site 1	1,160	13.5	44.7	1.5	62.8	Eutrophy
Szymon	154	1.1	2.9	1.1	62.9	Eutrophy

*SD* – Secchi disc visibility, *TSI* - trophic state index.

### Bacterial abundance and morphology

**Figure 3:**
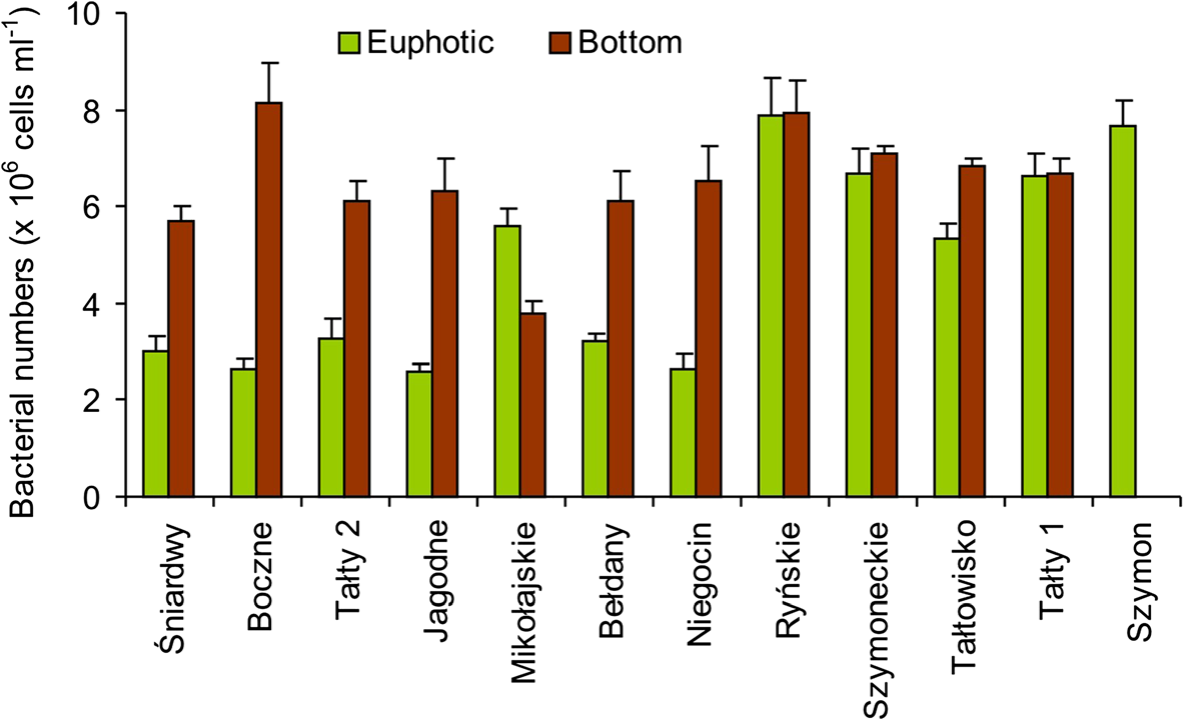
Total bacterial numbers in the euphotic zone (green columns) and bottom waters (brown columns) of the studied Mazurian lakes (mean valued and standard deviations of triplicate measurements).

In all of the studied lakes, except Lake Mikołajskie, bacterial numbers were considerably lower in the euphotic zone, where they ranged between 2.60 ± 0.13 and 7.88 ± 0.78 × 10^6^ cells ml^-1^, than in the bottom water layers (3.94 ± 0.37 – 8.13 ± 0.85 × 10^6^ cells ml^-1^) (Figure 3). The length of bacterial cells was shorter in the euphotic zone (0.75 ± 0.08 – 1.00 ± 0.03 μm) in comparison to the bottom layer (0.90 ± 0.04 – 1.17 ± 0.03 μm), except for Lake Śniardwy, where the bacterial cell length in the euphotic zone was the longest among the studied lakes. Similarly, the width of bacterial cells was narrower in the euphotic zone (0.385 ± 0.036 – 0.524 ± 0.045 μm) than in the bottom waters (0.473 ± 0.024 – 0.56 ± 0.017), except in two lakes, Śniardwy and Tałty 1 (data not shown). Statistical analysis revealed significant differences between the studied layers in both bacterial numbers (*p* < 0.05) and cell morphological parameters (Mann–Whitney *U*-test, *p* < 0.01).

### Bacterial activity

**Figure 4:**
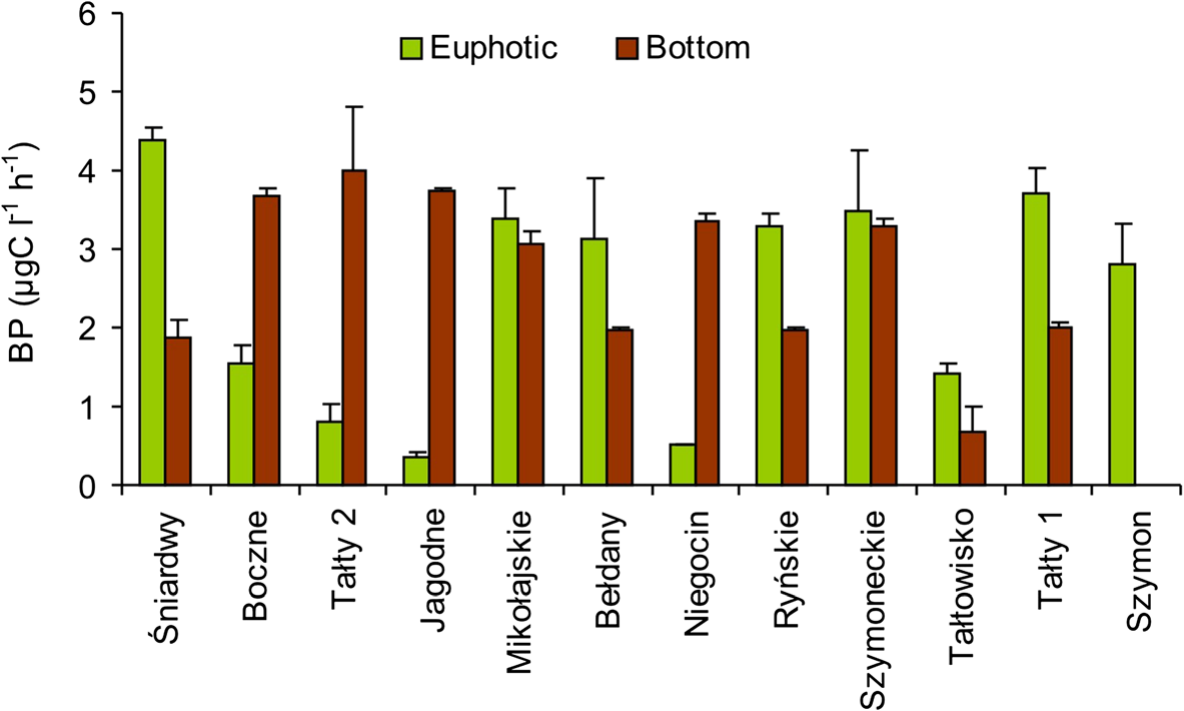
Bacterial secondary production (BP) in the euphotic zone (green columns) and bottom waters (brown columns) of the studied Mazurian lakes (mean valued and standard deviations of triplicate measurements).

In the euphotic zone of the studied lakes, bacterial secondary production (BP) ranged from 0.35 ± 0.06 to 4.40 ± 0.15 μg C l^-1^ h^-1^. In the bottom waters, BP was the lowest (0.67 ± 0.32 μg C l^-1^ h^-1^) in Lake Tałtowisko and the highest (4.01 ± 0.8 μg C l^-1^ h^-1^) in the highly eutrophic Lake Tałty 2 (Figure 4). Only in two lakes (Mikołajskie and Szymoneckie) were similar values of BP observed in both layers. In the meso-eutrophic lake and in lakes with higher eutrophic conditions (Bełdany, Ryńskie, Tałtowisko, Tałty 1), BP rates were about 2-times higher in the euphotic zone than in the bottom water layers. In less eutrophicated lakes (Boczne, Tałty 2, Jagodne, Niegocin), BP rates were markedly higher in the bottom waters and the differences between both the studied water layers were 5–7-fold and even, as in the case of Lake Jagodne, 11-fold. Statistically, however, these differences between layers were not significant (*p* > 0.05).

The percentage of respiring bacterial cells, examined by means of 5-cyano-2,3 ditotyl tetrazolium chloride reduction (CTC+), highly differentiated between the studied lakes and accounted for 1.5 to 15.4% in the euphotic zone and from 1.1 to 4.7% in the bottom waters of the total DAPI counts (Table 2). Hydrolytically active bacteria, examined by intracellular hydrolysis of fluorescein diacetate by non-specific esterases (EST+), accounted for 4.2 to 14.2% of the total DAPI bacterial numbers in the euphotic, and 2.7 to 11.3% in the bottom lake waters. The contribution of EST+ bacterial cells to the total DAPI numbers of bacteria was higher than CTC+ cells, especially at the bottom of the studied lakes. The percentage of bacteria with visibly integrated nucleoid (NuCC+) was the highest among the active bacteria examined by the applied approach. NuCC+ cells accounted for 50.5 to 97.3% in the euphotic zone and for 28.8 to 75.0% of the total DAPI counts in the bottom waters. In all of the studied lakes, the participation of CTC+, EST+ and NuCC+ bacteria was higher in the euphotic zone than at the bottom of the studied lakes, except for two lakes (Mikołajskie, Tałty 1) in the case of CTC+ and EST+ cells, and in three lakes (Śniardwy, Mikołajskie, Tałtowisko) in the case of NuCC+ cells. Statistical differences in the proportion of all fractions of active bacteria between both layers were significant (*p* < 0.05).

**Table 2:** Percentage contribution of bacterial cells with respiratory activity (CTC+), cellular esterase activity (EST+) and visible nucleoid (NuCC+) to the total bacterial DAPI-stained counts in the euphotic (E) and bottom (B) layers of the studied Mazurian lakes

**Lake**	**The percentage of metabolically active bacteria**					
	**CTC+**		**EST+**		**NuCC+**	
	**E**	**B**	**E**	**B**	**E**	**B**
Śniardwy	3.2	1.4	12.3	4.6	62.8	75.0
Boczne	1.9	1.1	12.7	3.6	70.8	37.1
Tałty 2	4.2	1.2	11.5	5.7	72.5	57.1
Jagodne	15.4	4.7	14.2	3.7	50.5	41.0
Mikołajskie	1.9	2.4	5.8	11.3	55.7	67.9
Bełdany	13.7	2.6	11.0	5.4	78.0	48.7
Niegocin	4.4	1.5	10.9	2.7	65.3	57.3
Ryńskie	8.8	1.1	7.0	3.5	76.4	38.4
Szymoneckie	5.6	2.6	4.2	2.8	79.7	28.8
Tałtowisko	12.2	2.3	8.4	5.2	55.8	72.6
Tałty 1	1.5	3.0	4.6	5.2	89.3	71.4
Szymon	6.7	-	6.0	-	97.3	-

### Nanoflagellate abundance

**Figure 5:**
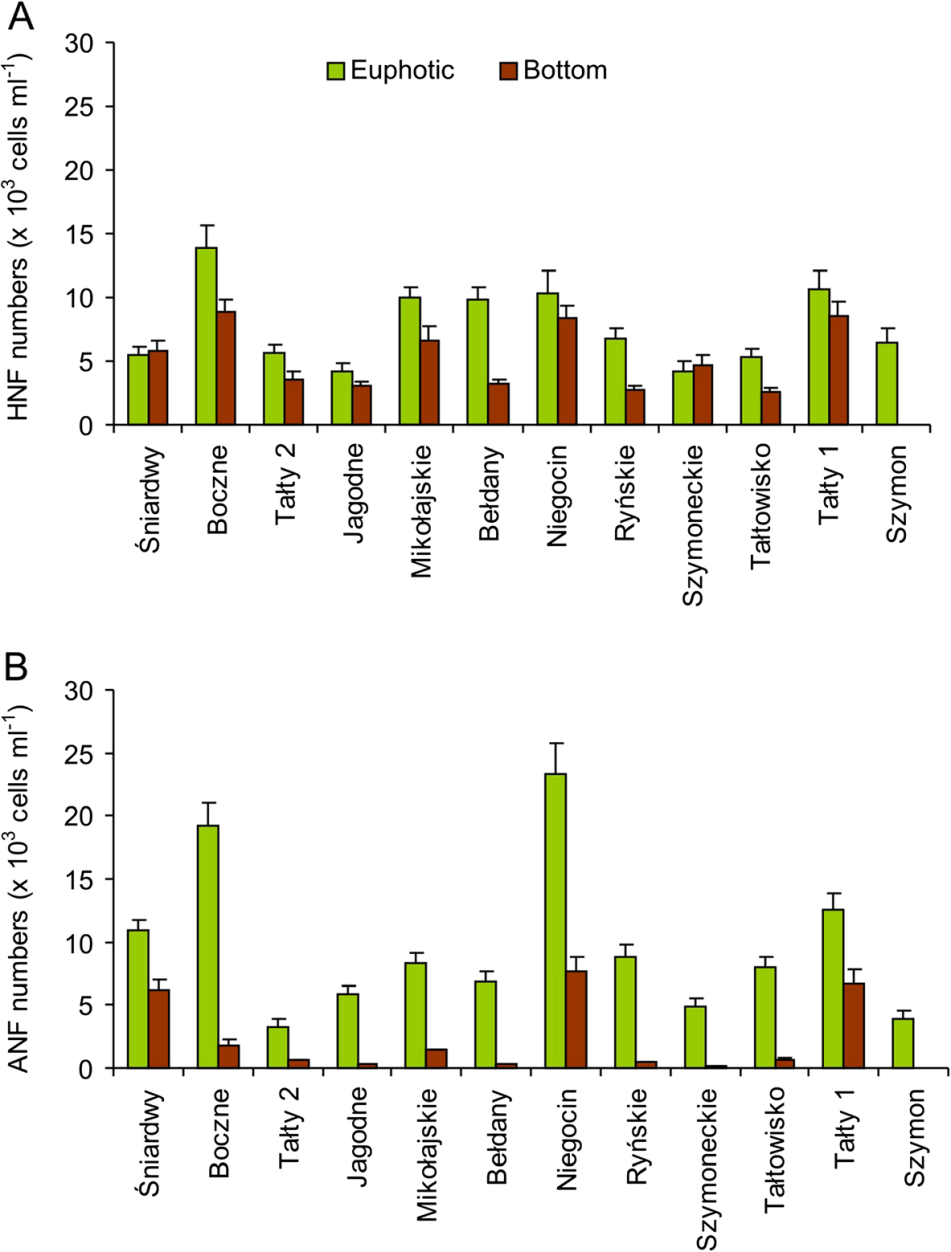
The numbers of (A) heterotrophic (HNF) and (B) autotrophic nanoflagellates (ANF) in the euphotic zone (green columns) and bottom waters (brown columns) of the studied Mazurian lakes (mean valued and standard deviations of triplicate measurements).

In the euphotic zone of the studied lakes, the total numbers of nanoflagellates ranged from 8.89 ± 0.98 to 33.56 ± 4.25 × 10^3^ cells ml^-1^. In the near-bottom water layer, the numbers were 1.4–5 times lower, ranging from 3.26 ± 0.36 to 16.04 ± 1.57 × 10^3^ cells ml^-1^ (Figure 5). The greatest differences between both lake water layers were observed in the lakes Bełdany, Ryńskie and Tałtowisko. Autotrophic cells of nanoflagellates (ANF) dominated in the euphotic zones of the majority of the studied lakes, while heterotrophic (HNF) nanoflagellates prevailed only in four of the studied lakes. In the bottom lake waters, HNF were the most abundant. It should be noted, however, that only in shallow Lake Śniardwy did ANF dominate in bottom waters, while both groups, ANF and HNF, were present in similar numbers in Lake Niegocin. In the majority of the studied lakes, except for lakes Szymoneckie and Śniardwy, the HNF numbers were decidedly higher in the euphotic zone than at the bottom of lakes (Figure 5A). Autotrophic nanoflagellates occurred in extremely low numbers at the bottom of the studied lakes and did not exceed one thousand cells ml^-1^, except in lakes with a depth of 9–11 m, where about 2 to 8 thousand cells per ml were observed (Figure 5B). The differences between the studied lake water layers for total nanoflagellates and both HNF and ANF numbers were statistically significant (*p* < 0.05).

### Ciliate abundance and composition

A total of 50 ciliate taxa were found in the studied lakes. Three ciliate orders were dominant in the euphotic zone. These were Oligotrichida, represented by *Rimostrombidium* species, Prostomatida (mainly small species from the genus *Urotricha*) and Hymenostomatida, mainly composed of *Histiobalantium bodamicum* Krainer and Müller, which constituted 36–56%, 36–51% and 37–42% of the total numbers, respectively. In the bottom waters, two ciliate orders, Oligotrichida (mainly *Tintinnidium* sp.) and Prostomatida (several species of *Urotricha*), dominated, accounting for 37–56% and 41–55% of the total numbers, respectively. The contribution of algivorous *Histiobalantium bodamicum* to the total ciliate numbers decreased clearly with depth, whereas the contribution of bacterivores such as scuticociliates (*Cinetochilum margaritaceum* (Ehrenberg), *Cyclidium glaucoma* Müller) and peritrichs (*Vorticella* spp.), as well as omnivorous haptorids *Mesodinium pulex* Stein, increased with depth.

**Figure 6:**
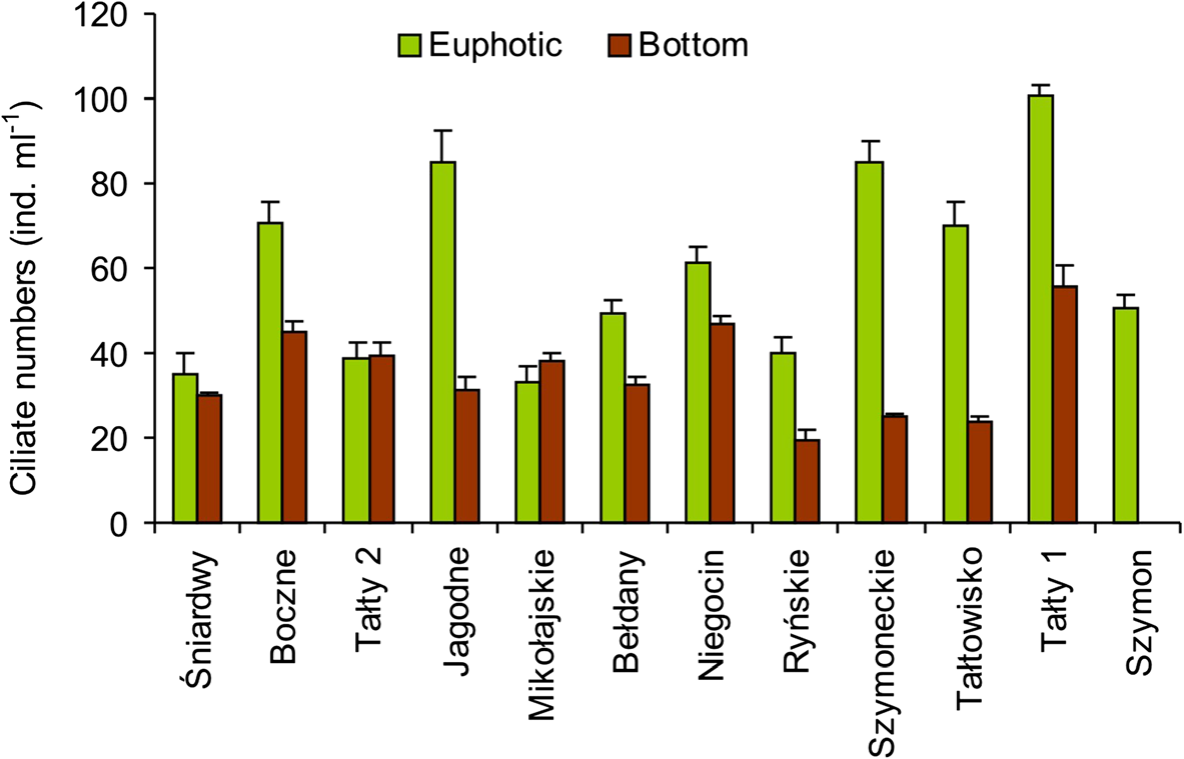
Total ciliate numbers in the euphotic zone (green columns) and bottom waters (brown columns) of the studied Mazurian lakes (mean valued and standard deviations of triplicate measurements).

Ciliate numbers were relatively high in the studied lakes, varying in a wide range from 32.9 **±** 4.2 to 100.5 ± 2.5 ind. ml^-1^ in the euphotic zone, and were 1.2–3.4 times higher than in the bottom lake waters, where they fluctuated from 19.2 **±** 2.7 to 55.8 ± 4.7 ind. ml^-1^ (Figure 6). Only in Lake Mikołajskie was the abundance of ciliates slightly higher in the profundal than in the euphotic zone. In one lake, Tałty 2, the ciliate abundance was almost identical in both layers. Statistically, differences between both layers were significant (*p* < 0.01).

### Relationships among microorganisms and environmental variables

Statistical analysis based on Pearson’s coefficient showed that in the euphotic zone, among the investigated groups of organisms, only bacterial numbers correlated positively with TSI of the studied lakes (r = 0.64, *p* = 0.024). Significant correlations were found between oligotrichs ciliates and chlorophyll *a* concentrations (r = 0.63, *p* = 0.03), as well as between nanoflagellates and dominating ciliate orders such as Oligotrichida (r = −0.59, *p* = 0.04) and Prostomatida (r = 0.62, *p* = 0.033) or dominating taxa such as *Urotricha* (r = 0.59, *p* = 0.044) and *Balanion planctonicum* (r = 0.66 *p* = 0.019). Among the staining methods used to examine bacterial cellular metabolic activity, only the percentage of EST + bacteria correlated negatively with TSI of lakes (r = −0.64, *p* = 0.026). Correlations between EST+ and NuCC+ cells and total phosphorus (TP) concentrations were also found; however, EST+ correlated negatively (r = −0.70, *p* = 0.011), while NuCC + correlated positively (r = 0.68, *p* = 0.014). Highly significant positive correlations were found between DOC concentrations and the numbers of CTC + cells (r = 0.82, *p* = 0.0011) and EST + cells (r = 0.83, *p* = 0.0009).

In the bottom waters, both nanoflagellates and ciliates correlated positively with chlorophyll *a* (r = 0.78 and r = 0.81, respectively, *p* < 0.01) and negatively with TP concentrations (r = −0.82 and r = −0.60, respectively, *p* < 0.05). In addition, highly significant positive correlations were found between oxygen concentrations and nanoflagellates (both total nanoflagellates, HNF and ANF) and ciliates (correlation coefficient varied between 0.71 and 0.86, *p* < 0.01), as well as between temperature and both groups of protists (r = from 0.73 to 0.90, *p* < 0.01). Significant positive correlations were recorded between ciliates and total numbers of nanoflagellates (r = 0.77, *p* = 0.005) and both their autotrophic (r = 0.62, *p* = 0.041) and heterotrophic forms (r = 0.82, *p* = 0.002).

**Figure 7:**
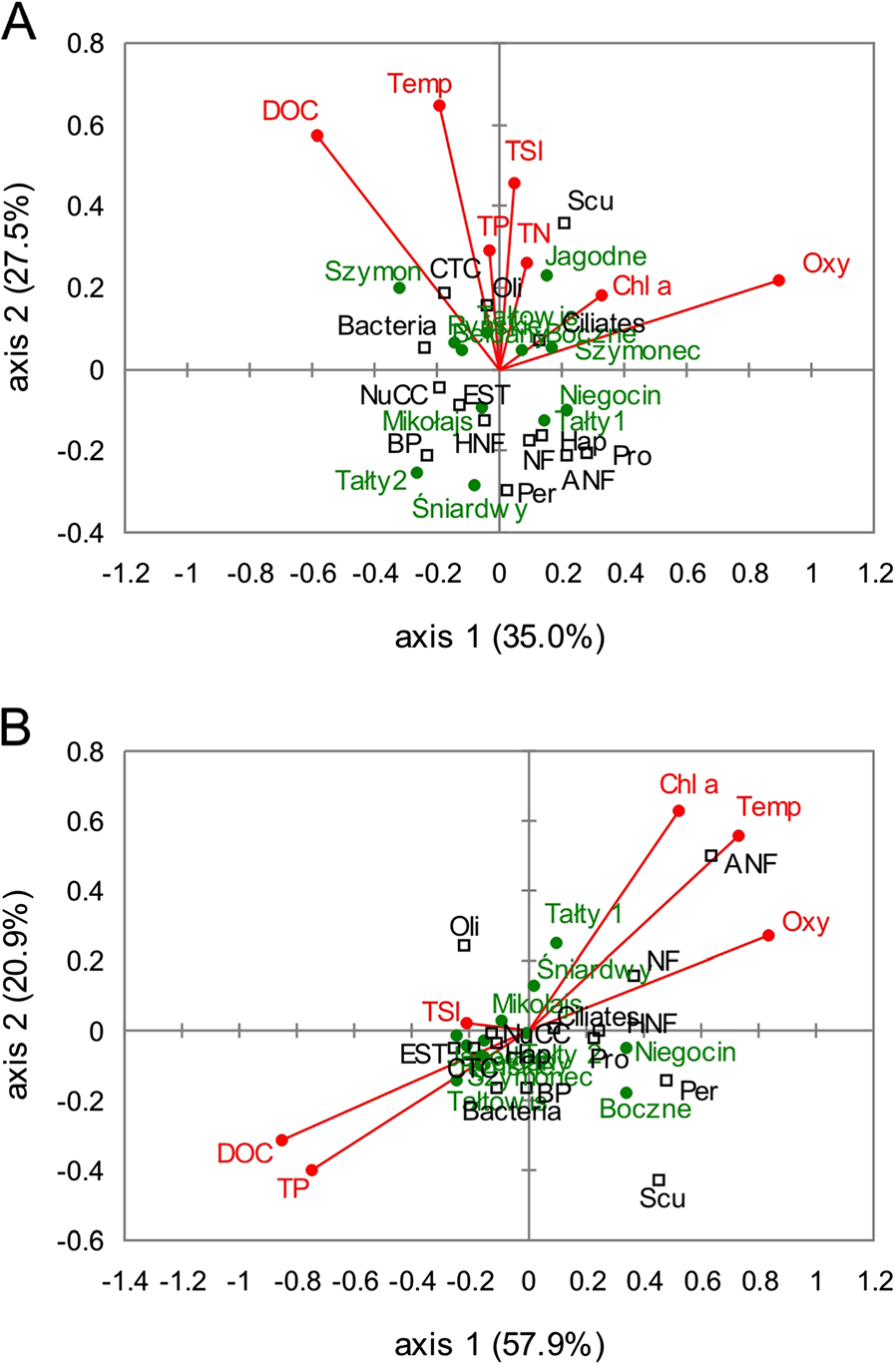
**Canonical Correspondence Analysis (CCA) ordination diagram showing the relationships between bacteria and protists and physico-chemical variables in the euphotic zone (A) and bottom waters (B) of the studied Mazurian lakes (Abiotic variables: Temp – temperature, Oxy – dissolved oxygen, DOC – dissolved organic carbon, TP – total phosphorus, TN – total nitrogen, Chl a – chlorophyll a, TSI – trophic state index.** Biotic variables: NF – nanoflagellates, HNF – heterotrophic nanoflagellates,ANF – autotrophic nanoflagellates; BP – bacterial production, CTC – respiring bacterial cells, EST – hydrolytically (esterase) active bacteria, NuCC – bacterial cells with visible integrated nucleoid; ciliate orders: Oli – Oligotrichida, Pro – Prostomatida, Hap – Haptorida, Scu – Scuticociliatida, Per – Peritrichida).

According to the CCA, the first two canonical axes together accounted for 62.5% and 78.8% of the variance in the microorganisms and the environmental variables in the euphotic and bottom water layers, respectively (Figure 7). In the euphotic zone, the first axis (35.0% of the variation) was associated with oxygen concentration. The second axis accounted for a further 27.5% of the variance and was related to temperature (Figure 7A). In the bottom waters, the first axis (57.9% of the variation) was associated with DOC, TP, oxygen, and temperature, while the second axis (20.9%) was positively correlated with chlorophyll *a* concentration (Figure 7B). The analysis revealed that in both water layers, ciliates were primarily associated with chlorophyll and nanoflagellates, while bacteria - with DOC and TP. In the bottom water layer, protists (especially nanoflagellates) were also correlated with temperature and oxygen concentration.

## Discussion

In the present study, we investigated bacteria and protists in lakes that were classified as eutrophic according to their trophic state index (TSI) values (56.9 to 62.8). Lake Śniardwy, with a TSI of 51.5 indicating meso/eutrophic properties, was included in the investigation because results of recent studies showed that this lake is undergoing the fast process of water eutrophication; bloom of the filamentous cyanobacteria is regularly observed during the summer [Chróst, unpublished data]. Moreover, bacterial and protistan numbers (similar to or even higher than in some eutrophic lakes) may indicate that this lake was more similar to the eutrophic than mesotrophic lakes. Our previous research [13,14], as well as this study, indicated that microbial parameters may be useful indicators of lake trophic status in addition to chemical parameters. Despite the similar trophic status, the studied lakes differed markedly in the abundances of bacteria and protists and domination structure of ciliate communities. The most visible differences (4-fold) were observed in nanoflagellates, especially in their autotrophic forms (7-fold differences).

Our studies were conducted at the end of April, which is a period of dynamic changes in the pelagic communities of plankton. These changes occur over a relatively short time and their role depends mostly on abiotic factors such as lake morphometry and climate variation [17]. This period differs from other seasons in many aspects. As shown by Kiersztyn et al. [18], TSI values estimated for the same lakes differ evidently between spring (April) and summer (July). In the present study, the TSI values of most of the studied lakes were higher than those estimated during the summer stratification period (unpublished data). Studies on the seasonal dynamics of planktonic organisms showed that bacteria e.g. [19], nanoflagellates [1,8,16,20,21] and ciliates [8,10,22], reach usually maximal abundances and/or biomass during spring period, especially in eutrophic/hypereutrophic lakes. At this time, they are probably controlled by different environmental and biotic factors than in summer and/or autumn. According to Šimek et al. [23], the bottom-up control may be more important during spring and top-down control during summer.

In the euphotic zone, we found high numbers of nanoflagellates (8.89 to 33.56 × 10^3^ cells ml^-1^; mean 17.36) and ciliates (32.9 to 100.5 ind. ml^-1^; mean 59.9) that were 2–3-times higher than those noted by Chróst et al. [14] during the summer stratification period. In contrast, bacterial abundances were relatively low (2.60 to 7.88 × 10^6^ cells; mean 4.76); 3–4 times lower in comparison to the results of studies by other authors that were conducted in the same lakes [14,24]. In the euphotic zone, among the investigated groups of organisms, only bacterial numbers increased with the increasing trophic status of the studied lakes. This fact may indicate that not all microorganisms were affected in the same way by eutrophication [21].

Season strongly affects the relationships between different planktonic groups of organisms [8,16]. Sanders et al. [25] found that the top-down control (predation) seems to be more important in regulating prokaryotic abundances in eutrophic systems than in oligotrophic systems in which bottom-up control (substrate and nutrient availability) plays a significant role. It is well known that protists, especially heterotrophic nanoflagellates, are the most effective consumers of bacteria [1,26]. However, depending on the trophic status of lake ciliates, autotrophic nanoflagellates, rotifers and cladocerans can also be important bacterivores [26]. In addition, the role of protists in controlling bacterial abundances is altered in different seasons and years in the same reservoir [1,2]. According to several authors [8,27,28], the abundance of bacteria and protists often shows a lack of coupling across trophic gradients, especially in more eutrophic environments during spring.

In the present study, we did not find any significant correlations between bacterial and protistan numbers, indicating that both nanoflagellates and ciliates were not a major factor controlling bacterial abundance in eutrophic Mazurian lakes during spring. This conclusion is supported by the results of the CCA analysis, which did not show clear relationships between these groups of organisms, both in the euphotic and bottom water layers (Figure 7). In contrast, studies by Koton-Czarnecka and Chróst [29], which were conducted in the pelagial of ten Mazurian lakes, showed that grazing on bacteria was more efficient in April than in July and protistan grazing removed more than 31% of the bacterial biomass. Ciliates play a significant role as consumers of small algae, autotrophic picoplankton and nanoflagellates. They may also be active bacterivores, particularly in eutrophic lakes, where bacterial densities are sufficient to maintain ciliate populations [1,30]. This study showed that the ciliate community was dominated mainly by small prostomatids *Balanion planctonicum* and *Urotricha* spp. (mainly *U. furcata*) feeding on bacteria and algae (including flagellates), small oligotrichs *Rimostrombidium humile*, feeding mainly on algae (including diatoms), and larger (40–60 μm in size) scuticociliate *Histiobalantium bodamicum,* feeding mostly on algae (e.g. cryptophyte *Rhodomonas* sp.) [31]. Significant correlations between oligotrichs ciliates and chlorophyll *a* concentrations, as well as between nanoflagellates and dominating ciliate orders such as Oligotrichida and Prostomatida or dominating taxa such as *Urotricha* and *Balanion planctonicum*, may indicate that ciliates were probably associated with spring phytoplankton bloom and played an important role as consumers of small algae and nanoflagellates. All of these facts and a lack of coupling between ciliates and bacteria show that ciliates were probably more important with respect to algivory than bacterivory. A similar conclusion was found by other authors e.g. [10,11,31,32], who suggested that high abundances of small algivorous ciliate taxa in spring (April) in dimictic lakes are related to the mass development of small algae (including flagellates *Rhodomonas*, *Cryptomonas* and diatoms) and may be the most important grazers of the spring phytoplankton bloom.

Bacteria differed not only in cell-size and community composition, but mainly varied in metabolic activities in the studied lakes. Studies performed in both marine and freshwater environments, using different staining techniques, showed that only a small fraction (generally less than 20%) of the total bacterial microscopic counts is metabolically active e.g. [33-36]. Factors regulating the numbers and proportion of metabolically active bacteria (temperature, viruses, nutrients, organic substrates and grazing by flagellates and ciliates) are not fully understood [37] and vary with a very short time scale [38]. Many studies, mostly experimental, have shown that the low percentage of active cells was the consequence of strong effective and continuous protozoan grazing pressure e.g. [37,39,40]. Among protists, HNF plays the crucial role in shaping abundance, biomass and morphology of the active fraction of bacteria [37,41,42].

In this study, three staining methods (CTC, EST, and NuCC) were used to determine different aspects of the cellular state or metabolic activity of bacteria. The percentage of bacteria with an active electron transport system (CTC+ cells) was the lowest (1.1–15.4% of the total DAPI counts) among the studied fractions of activity. This confirms the observations of Sherr et al. [43], who showed that CTC+ cells represent a varied active fraction of the total bacterial abundance. A relatively low percentage of CTC+ cells (0.1 to 9.5% of the total counts) was found by other authors [3,39]. Higher proportions of these cells (about 20–30%) were recorded by Berman et al. [34], Søndergaard and Danielsen [38], and del Giorgio and Scarborough [44].

There are not many data published on bacteria with cellular esterase activity (EST+). As shown by Schumann et al. [35], the percentage of these cells ranged from 2 to 24% depending on the water systems and was higher in estuaries (mean 12%) than in freshwater (9%) and the Baltic Sea (5%). In this study, the share of EST+ bacteria was low in all lakes (2.7 to 14.2% of the total counts) and was comparable to the CTC+ bacterial cells.

Comparison of the major methods used to assess active bacterial cells showed that the lowest percentage of active cells in freshwaters were recorded with the CTC and EST assay in contrast to NuCC (nucleoid-containing bacteria) and MEM+ (cells with an intact membrane) methods [34]. According to the literature data, NuCC may constitute 37–90% of total counts [41], while MEM+ cells 42–90% [3,35] of the total bacterial counts, with the maximal percentage in spring [3]. In the present study, the share of NuCC+ in the total numbers was relatively high and did not differ markedly between individual lakes (50.5–97.3% of the total counts in the euphotic zone); this is in contrast to CTC+ cells, which showed 10–fold differences.

Several authors indicated that the abundance and proportion of active cells (mostly CTC+) increased with increasing lake productivity during the summer period [38,44]. However, little information is available on the influence of lake trophic status on other fractions of active bacterial cells (EST+, NuCC+, MEM+). Adamczewski [24] showed that in Mazurian lakes, the share of active bacteria to the total bacterial numbers and biomass (4–27%) markedly differed among the studied lakes, and did not show a relationship with lake water trophic status, either in the surface or bottom water layers. Moreover, mesotrophic lakes were characterized by a considerably higher contribution of MEM+ than eutrophic lakes. Also, Chróst et al. [14] indicated that the contribution of MEM+ bacteria to the total bacterial numbers (16–24%) and bacterial biomass (9–14%) did not respond strongly to the trophic status of lakes. In our study, only the percentage of EST+ bacteria correlated negatively with TSI of lakes. Correlations between EST+ and NuCC+ cells and total phosphorus (TP) concentrations were also found; however, EST+ correlated negatively, while NuCC+ correlated positively. Highly significant positive correlations were found between DOC concentrations and the numbers of CTC+ cells and EST+ cells, which may suggest that nutrients and organic substrates were important factors controlling the active metabolism of bacteria. Our results are in agreement with other studies [33-35]. We did not find any correlation between nanoflagellates, both autotrophic and heterotrophic, and numbers of active cells of bacteria. This may imply that the bottom-up control by substrates was more important than top-down grazing control by flagellates in regulating the abundance of metabolically active bacterial cells in eutrophic lakes during the spring period. The major source of labile carbon (i.e. easily accessible to bacteria, e.g. proteins) is phytoplankton primary production. During the spring, a large portion of organic matter in lake waters is of allochthonous origin because of the surface runoff from surrounding watershed [13]. The allochthonic matter is poor in proteins, but rich in aromatic cyclic compounds (e.g. polyphenols) that are resistant to microbial degradation; this is an argument supporting a bottom-up control mechanism. Literature data show that ciliates may also be a factor determining active bacterial cells [34,45]. However, ciliates are not selective and feed on all available bacterial-food. In addition, as mentioned above, ciliates preferentially consumed algae than bacteria in eutrophic Mazurian lakes.

This study was conducted at the end of April, which is the initial phase of stratification and phytoplankton bloom. We did not observe a vertical uniformity in temperature; however, in most lakes the temperature was only slightly lower at the bottom (2.9–8.3°C) than in the euphotic zone (7.3–13.3°C). Thus, plankton organisms were not evenly distributed between the two layers, euphotic and bottom, as during the spring lake water circulation, and did not show a typical distribution in the entire water column as during the summer stratification period [46]. However, studies performed by Berdjeb et al. [47] in deep pre-alpine lakes, which differ in trophic status, revealed significant differences in the bacterial community structure with depth, regardless of whether the water column was mixed or stratified. It is known that many species of flagellates and ciliates may migrate diurnally throughout the water columns due to top-down grazing pressure by zooplankton e.g. [48,49]. Thus, microbial communities may vary more with water column depth than through a season, especially during periods of increasing thermal stratification [47,50,51].

In the bottom waters, ciliate (19.2–55.8 ind. ml ^-1^ – values 2–3-times lower than in the euphotic zone) and nanoflagellate numbers (3.30–6.04 × 10^3^ cells ml^-1^ – values 2–5-times lower than in the euphotic zone) were relatively high. The numbers of HNF were more or less evenly distributed between the two studied zones, whereas ANF occurred in relatively low numbers in comparison to the euphotic zone. Both nanoflagellates and ciliates correlated positively with chlorophyll *a* and negatively with TP concentrations. In addition, highly significant positive correlations were found between oxygen concentrations and nanoflagellates (both total nanoflagellates, HNF and ANF) and ciliates, as well as between temperature and both groups of protists. All of these correlations may indicate that trophic conditions and environmental physical parameters were important in controlling protozoan communities at the bottom level. The results of CCA analysis provided similar conclusion (Figure 7). Similarly as in the euphotic zone, algivorous ciliates (*Tintinnidium* sp. and *Urotricha* spp.) dominated. Small bacterivorous scuticociliates and omnivorous *Mesodinium* sp. were more abundant in the deeper water and their occurrence was mainly associated with a large amount of detritus-bound bacterial food [22]. Significant positive correlations between ciliates and total numbers of nanoflagellates and both their autotrophic and heterotrophic forms suggest the mutual relationship between these components.

Bacterial numbers, biomass, cell-size, and secondary production were generally higher in the bottom lake water than in the euphotic zone. Only the contributions of metabolically active cells of bacteria to the total bacterial counts were lower in the bottom water layer, suggesting that metabolically active cells were probably under high grazing pressure. Similar results were found by Berman et al. [34], who showed that the mean percentage contribution of metabolically active bacterial cells was lower at 1 m depth (CTC – 4.1%, NuCC – 7.2%, MEM + − 7.6%) than at 38 m depth (CTC – 6.5%, NuCC – 9.7%, MEM + − 8.8%) in meso-eutrophic Lake Kinneret. In contrast, Adamczewski [24] showed that the maximal share of MEM+ in total bacterial numbers was higher in the hypolimnion (26.3%) than in the epilimnion (18.8%) in 17 Mazurian lakes of different trophies. The author suggested that the higher proportion of metabolically active cells in anoxic water at the bottom of lake indicated lower grazing pressure by bacterivorous protists in comparison to the surface water layer of the studied lakes where the grazing of heterotrophic nanoflagellates leads to a lower abundance and biomass of metabolically active bacteria. In the present study, bacteria, both their total numbers and metabolically active cells, did not correlate with the studied abiotic (DOC, TP, temperature, oxygen) and biotic (chlorophyll, nanoflagellates, ciliates) parameters. However, besides the above-mentioned factors regulating bacterial communities, “inside-control” by viral lysis exists e.g., [37,47,52]. As shown by Berdjeb et al. [47], the magnitude of this mechanism may vary by depth. The authors indicated that top-down regulation by flagellates together with ciliates or viruses was important in controlling bacterial community structure only in the hypolimnion, in contrast to the epilimnion, where bacteria were dependent mainly on bottom-up factors. In our study, the lack of coupling between bacteria and the studied parameters may suggest that infection by viral lysis probably affected bacteria at the bottom of eutrophic Mazurian lakes during the spring period.

## Conclusions

During the spring period, eutrophic Mazurian lakes were characterized by high nanoflagellate and ciliate abundances, relatively low bacterial numbers, more or less uniform ciliate community composition in both euphotic and bottom layers, as well as a lack of significant relationships between bacterial and protistan numbers. In the euphotic zone, the concentrations of total phosphorus (TP) and dissolved organic carbon (DOC) significantly controlled (bottom-up control mechanism) the bacterial abundance and metabolic activity. None of the studied physico-chemical and biological factors influenced the bacterial abundance and cellular metabolic activity in the bottom lake waters. Our studies suggest that, in this water layer of lakes, bacterial communities were probably simultaneously affected by both regulatory mechanisms, i.e. bottom-up and top-down controls, and/or influenced by an “inside control” mechanism, probably by viral lysis.

In both layers, the availability of appropriate food (algae and nanoflagellates but not bacteria) was probably a major factor determining ciliate abundance and composition. At the bottom of the studied lakes, both groups of protists appeared to also be influenced by physical and chemical variables such as dissolved oxygen concentration, temperature and concentration of total phosphorus.

## Methods

### Study area and sampling

**Figure 8:**
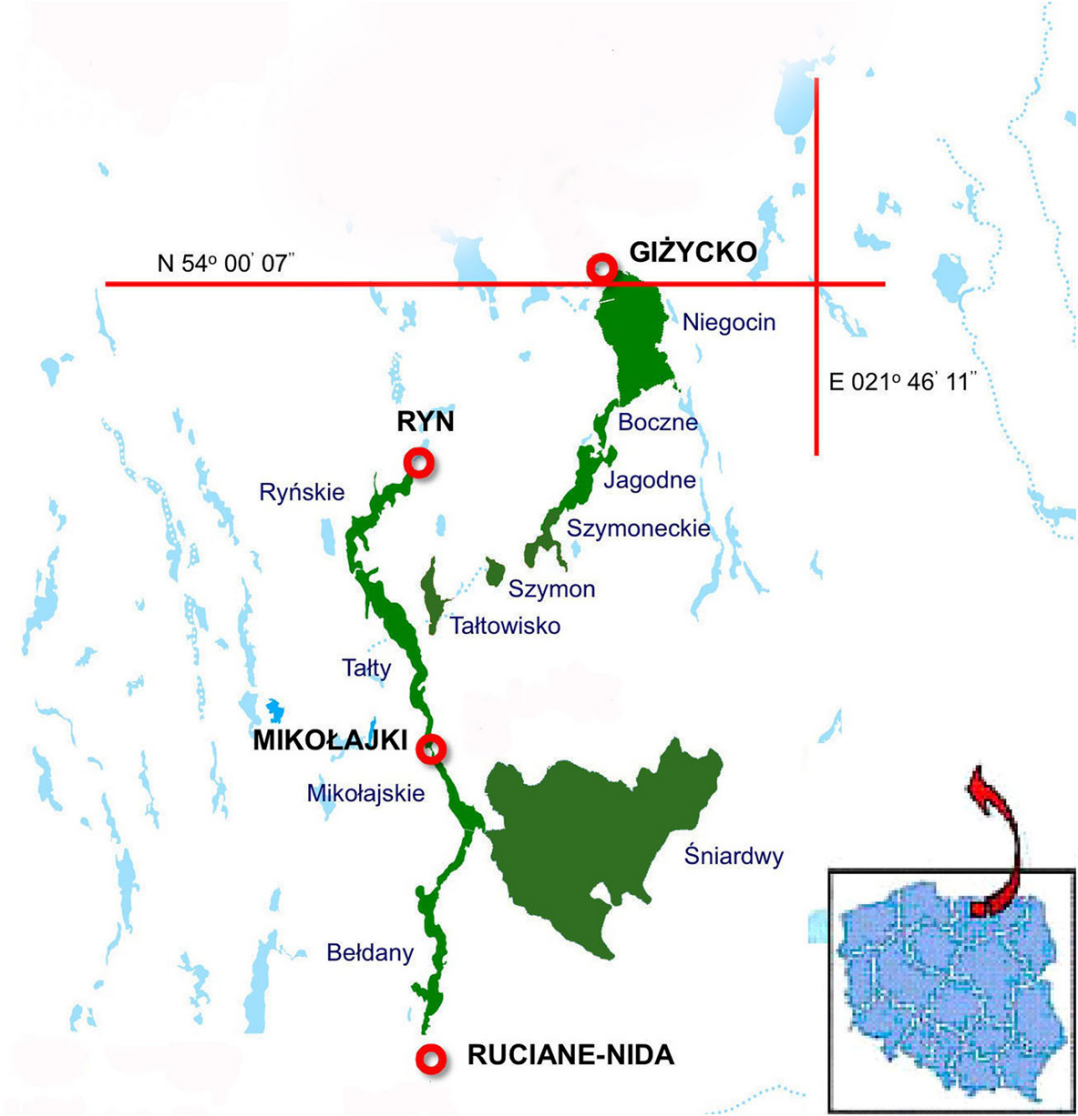
Location of the studied lakes (Mazurian Lake District, Poland).

The studies were conducted in the pelagial zone of eleven Mazurian lakes (north-eastern Poland) (Figure 8). In Lake Tałty, samples were collected from 2 sites (Tałty 1 and Tałty 2). Basic morphological parameters of the studied lakes are presented in Table 1. Trophic state index (TSI) of lakes, calculated from chlorophyll *a* and total phosphorus (TP) concentrations, and Secchi disc visibility (SD) according to Carlson [53], indicated that all of the studied lakes were eutrophic (TSI from 56.9 to 62.9), except Lake Śniardwy, with a TSI of 51.5 that indicated a meso/eutrophic characteristic (Table 1). All of the studied lakes, except for the shallow lakes Szymon and Śniardwy, are dimictic with summer distinct thermal stratification. Samples of water were collected in the spring (between 22 and 28 April). Triplicate water samples were taken in the pelagial zone at the deepest part of the lakes, from the euphotic layer (assumed to be equal to double Secchi disc visibility) and from the profundal of lakes 1 m above bottom sediments. Water samples from the euphotic zone were collected at 0.5 m intervals and mixed (v/v) to obtain one integrated sample of lake water.

### Physical and chemical analyses

Water temperature, pH, conductivity and oxygen concentration were measured *in situ* at 0.5 m depth intervals with an YSI 6600-meter (Yellow Spring Instruments, USA). Chlorophyll *a* (Chl *a*), extracted with 98% acetone, was measured using a TD-700 (Turner Design, USA) fluorimeter [54]. Total phosphorus (TP) concentration was determined spectrophotometrically according to Koroleff [55]. The sum of Kjeldahl nitrogen (analyzed in unfiltered water samples by the standard Kjeldahl procedure) and nitrate-nitrogen (analyzed by the phenyldisulphonic method in water filtered through the Whatman GF/C filters) represented total nitrogen (TN). The concentration of dissolved organic carbon (DOC) was determined in water samples filtered through 0.2 μm pore-size polycarbonate membrane filters (Millipore) using a Shimadzu TOC 5050 carbon analyzer.

### Bacterial abundance and morphology

Water samples preserved with formaldehyde (final concentration 2%), stained with DAPI (final concentration 1 μg ml^-1^) were filtered through 0.2 μm pore-size black polycarbonate membrane filters (Nucleopore) and enumerated by epifluorescence microscope Nikon ECLIPSE E 400 [56]. Bacterial cell parameters such as length (CL) and width (CW) were performed by means of Nikon Image System (NIS) software analysis.

### Bacterial activity

Three different methods were used to assess the share of metabolically active bacteria in total DAPI-stained bacterial cell numbers. Respiring bacterial cells, with an active electron transport system, were identified with CTC (5-cyano-2,3-ditolyl tetrazolium chloride) according to Rodriquez et al. [57]. Nucleoid-containing bacteria (NuCC) were determined in formalin-preserved, DAPI stained, and hot (60°C) isopropanol-rinsed samples [58]. Esterase-active bacteria were determined with 5-chloromethylfluorescein diacetate (CellTracker™ Green CMFDA) according to Haugland [59]. Bacterial secondary production (BP) was determined by the [^3^H]-thymidine method [60].

### Nanoflagellate abundance

Samples were fixed with formaldehyde (2% final concentration), stained with DAPI (final concentration 1 μg ml^-1^), filtered through 1.0 μm pore-size black polycarbonate membrane filters (Millipore) and enumerated by epifluorescence microscope Nikon Optiphot 2 [56]. Autotrophic (ANF) and heterotrophic nanoflagellates (HNF) were differentiated on the basis of chlorophyll *a* autofluorescence.

### Ciliate abundance and composition

Water samples were fixed with Lugol’s solution and examined by light microscopy (Nikon Optiphot 2). Species composition and measurements were determined from living material using a phase contrast, immersion, and stains for the nuclei and food vacuoles. Species identifications of ciliates were based mainly on Foissner et al. [61].

### Statistical analyses

Statistical analyses of results were carried out using the STATISTICA software package. The percentage data (proportions of metabolically active bacteria) were arcsine-transformed. The non-parametric Mann–Whitney *U*-test was used to analyze any differences in the studied parameters between zones. Relationships among the studied parameters were calculated using Pearson’s correlation analysis (n = 12). In addition, the influence of the physico-chemical and biological parameters on bacterial and protistan abundances was determined by the Canonical Correspondence Analysis (CCA) using the program XLSTAT-ADA 2013 (Addinsoft).

## Competing interests

The authors declare that they have no competing interests.

## Authors’ contributions

KK identified protists and drafted the manuscript. AG counted DAPI-stained bacteria and analyzed bacterial cell-size, BK analyzed microscopically metabolically active bacteria, RJCH measured bacterial secondary production and revised a draft version of a manuscript. All authors read and approved the final manuscript.
